# Picric Acid in the Treatment of Burns

**Published:** 1896-05-16

**Authors:** George Johnson


### PICRIC ACID IN THE TREATMENT OF BURNS.

By Sir George Johnson, M.D., F.R.C.P., F.R.S.

I have read with much interest the extract from the *Gazette des Hopitaux* on the above subject in your issue of May 2nd. Reference is therein made to the non-explosibility of uncombined picric acid. I once inadvertently put this to the test by a very conclusive experiment. On one occasion, intending to make a saturated solution of the acid, which I am constantly using as an invaluable test for albumen and sugar in the urine, I placed sixty-five grains of the crystals in a porcelain capsule over a Bunsen gas burner. I then sat down to read a paper, but in a few minutes I was disturbed by very acrid fumes from the capsule, when I found, to my surprise, that I had omitted to add the twelve ounces of water which it had been my intention to add. The result of this exposure of the dry crystals to a high temperature was that they were gradually being burned away without the slightest explosive action. Uncombined picric acid is no more explosive either by heat or by percussion than uncombined charcoal.

In the directions for the use of the acid in the treatment of burns the author advises " to avoid touching healthy parts of the skin or clothing with the application, as a dark yellow stain is left wherever the acid touches." It is true that clothing is indelibly stained by the acid ; but the stain on the skin, unlike that made by strong nitric acid, is readily removed by soap and water, as I can testify from ample experience.

As picric acid promises to be a valuable application for burns, no one need be deterred from using it by an unfounded fear of its explosiveness, or of its application leaving a permanent or durable stain upon healthy skin. Some years ago, when every reasonable and unreasonable objection was being urged against the use of picric acid as a test, an anonymous doctor wrote to one of the journals protesting against the employment of an agent so explosive that enough might be carried in the waistcoat pocket to blow the body to pieces. This sapient doctor, like many others, of whom it has been said that they were "never so dogmatic as when their knowledge was small," had not learned to distinguish between picric acid and its combination with potassium and other bases, a mistake not less than that of supposing that powdered charcoal and gunpowder are equally explosive.

